# Inactivation of *Rbx1* E3 ligase suppresses *Kras^G12D^*‐driven lung tumorigenesis

**DOI:** 10.1002/mco2.332

**Published:** 2023-07-18

**Authors:** Yanan Li, Di Wu, Haomin Li, Yi Sun

**Affiliations:** ^1^ Cancer Institute (Key Laboratory of Cancer Prevention and Intervention, China National Ministry of Education) of the Second Affiliated Hospital, and Institute of Translational Medicine Zhejiang University School of Medicine Hangzhou China; ^2^ Research Center for Life Science and Human Health of Binjiang Institute Zhejiang University Hangzhou China; ^3^ Children's Hospital Zhejiang University School of Medicine Hangzhou Zhejiang China

Dear editor,

Cullin‐RING ligase (CRL) is a multicomponent E3 ligase, consisting of a scaffold protein Cullin (with 8 family members), an adaptor protein (with many family members), a substrate receptor (with many family members), and a catalytic component with two members, RING‐box 1 (RBX1/ROC1) or RING‐box 2 (RBX2/ROC2/RNF7/SAG). As the scaffold protein, the Cullin binds at its N‐terminus to adaptor‐substrate receptor proteins and at its C‐terminus to the RING protein, which binds to ubiquitin‐loaded E2 to catalyze the ubiquitin transfer to a substrate. CRL is the largest E3 family, responsible for ubiquitylation of about ∼20% of cellular proteins doomed to proteasome degradation by proteasome. Of two RING family members, RBX1 complexes with Cullins 1−4, whereas RBX2/SAG complexes with Cullin 5 to form active CRLs 1−4 and CRL 5, respectively. By promoting ubiquitylation and degradation of short‐lived cellular signal proteins, CRLs regulates many key biological processes, including cell cycle progression, apoptosis, stress responsiveness, DNA damage and repair, DNA replication, viral infection, and tumorigenesis.^1^

RBX1 is a constitutively expressed and evolutionarily conserved RING component of CRLs 1−4. Our previously study, using genetic modified mouse model, showed that *Rbx1* total knockout resulted in early embryonic lethal at E6.5,^2^ indicating that Rbx1 is essential for mouse embryogenesis. Likewise, *Rbx1* silencing in *C*. *elegans* also induced lethality during development of embryos and in adulthood by triggering DNA damage response in intestinal cells via accumulated CDT1.^3^ Furthermore, using the in vitro cell culture models, we found that *RBX1* was overexpressed in lung tumor cell lines, and *RBX1* knockdown suppressed the growth of lung cancer cells by triggering apoptosis, G2/M arrest, DNA damage and senescence.^4^ Thus, RBX1 appears to be a growth essential gene in prolifcation and survival of lung cancer cells. However, it is unknown whether and how RBX1 affects the in vivo lung tumorigenesis.

Our previous study using immune‐histochemical staining with limited samples showed that RBX1 is overexpressed in lung cancer tissues.^4^ To extend this study, we searched the database (gepia.cancer‐pku.cn) with large sample collection, and found that *RBX1* was indeed overexpressed in lung adenocarcinoma tissues, as compared to normal tissues (Figure 1A). However, this association study did not distinguish whether *RBX1* overexpression is the cause or consequence of lung tumorigenesis (Supporting information).

**FIGURE 1:**
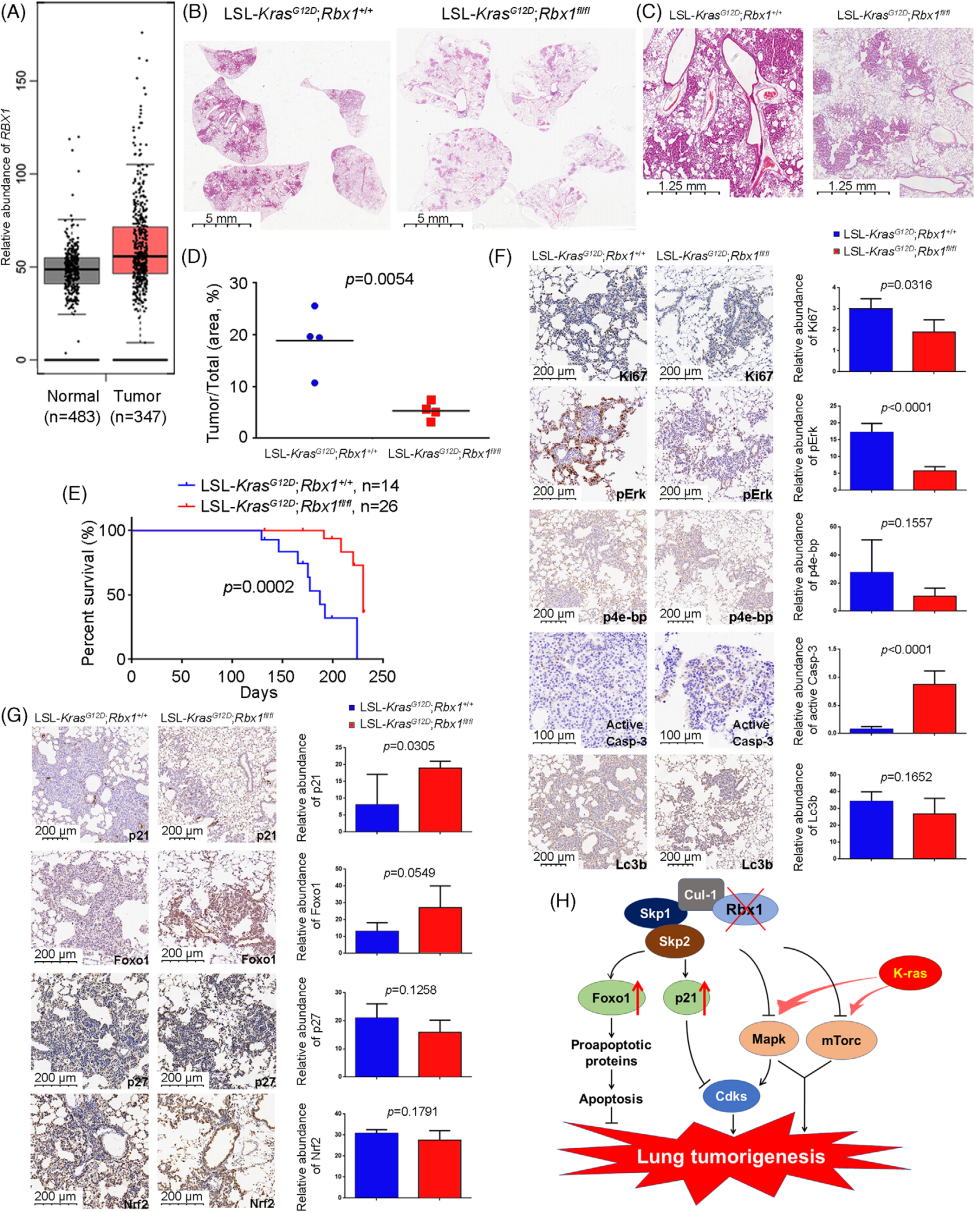
*Rbx1* inactivation suppressed lung tumorigenesis driven by *Kras^G12D^*. (A) *RBX1* mRNA expression in lung tumor and normal tissues. (B) Overall image of morphological observation of lung lobes from mice with indicated genotypes, 12 weeks after Ad‐Cre administration to activate *Kras^G12D^* and/or inactivate *Rbx1*. (C) Enlarged image of morphological observation of lung tissue from mice with indicated genotypes, 12 weeks after Ad‐Cre administration to activate *Kras^G12D^* and/or inactivate *Rbx1*. (D) Ratios of the areas of tumor tissues vs. total lung tissues (*n* = 4). Quantification was performed by Image J. Statistical analysis by Student *t* test. (E) Survival curve of *LSL*‐*Kras^G12D^*; *Rbx1^+^* ^/^ *^+^* control mice (*n* = 14) versus *LSL*‐*Kras^G12D^*;*Rbx1^fl^* ^/^ *^fl^* mice (*n* = 26) for up to 231 days after administration of Ad‐Cre to activate *Kras^G12D^* (control) as well as inactivate *Rbx1* (experiment). Statistical analysis by log‐rank test. (F) Reduced proliferation and increased apoptosis in *Rbx1*‐deleted lung tumor tissues. The lung tissues from mice of two genotypes were stained with indicated antibodies. Quantification was performed by Image J. Data were summarized from 5 mouse tumors. Statistical analysis by Student *t* test. (G) Accumulation of Rbx1‐substrates upon Rbx1‐deletion in lung tumor tissues. The lung tissues from mice of two genotypes were stained with indicated antibodies. Quantification was performed by Image J. Data were summarized from 5 mouse tumors. Statistical analysis by Student *t* test. (H) Working model of inactivation of Rbx1 E3 ligase suppresses *Kras^G12D^*‐driven lung tumorigenesis.

To this end, we investigated the role of *Rbx1* in lung tumorigenesis, using a well‐established *LSL*‐*Kras^G12D^* mouse lung tumor model, in which mutant *Kras^G12D^* is activated by Ad‐Cre virus infection to remove the upstream STOP fragment (LSL). We first crossed *Rbx1^fl^* ^/^ *^fl^* mice with LSL‐*Kras^G12D^* mice to generate mice with the genotypes of LSL‐*Kras^G12D^*;*Rbx1^+^* ^/^ *^+^* (control group), and LSL‐*Kras^G12D^*;*Rbx1^fl^* ^/^ *^fl^* (compound mice, experimental group). The mice at age of 8−10 weeks were intratracheally administrated with Ad‐Cre to activate *Kras^G12D^* alone (control), or to activate *Kras^G12D^* with simultaneously inactivate *Rbx1* (experiment). We harvested mouse lung tissues 12 weeks post Ad‐Cre administration and performed hematoxylin and eosin (H&E) staining for morphological observations. The results showed that the number of tumors and the size of tumors are both reduced in LSL‐*Kras* ^G12D^;*Rbx1^fl^* ^/^ *^fl^*, as compared to the LSL‐*Kras^G12D^*;*Rbx1^+^* ^/^ *^+^* mice control (Figure 1B–D).

To investigate whether tumor suppression by *Rbx1* deletion can be reflected by an extension in mouse life‐span, we determined the mouse survival after Ad‐Cre administration. Indeed, while 100% of control mice died 224 days (32 weeks) post Ad‐Cre administration, as expected, the death rate in compound mice were 73.5% at 32 weeks, which is significantly longer with a *p*‐value = 0.0002 (Figure 1E). The results clearly demonstrated that *Rbx1*‐deletion significantly reduced lung tumor burden and extended mouse life‐span. Thus, Rbx1 cooperates with Kras^G12D^ to promote lung tumorigenesis.

To determine the nature of tumor suppression caused by *Rbx1*‐deletion, we utilized the immunohistochemical (IHC) staining analysis and found that in hyperplasia/adenomas tissues from LSL‐*Kras^G12D^*;*Rbx1^fl^* ^/^ *^fl^* mice, the staining/levels of Ki67 and pErk were significantly reduced, along with p4e‐bp1, although it did not reach the statistically significant level due to sample variations (Figure 1F). An increased level of active Caspase‐3, but no change in the level of Lc3b was also observed (Figure 1F). Thus, it appears that *Rbx1*‐deletion inhibits proliferation and induces apoptosis likely via inactivating Mapk and mTorc pathways, and activating apoptosis pathway in *Kras^G12D^* lung tumor model.

Next, we measured potential accumulation of direct substrates of Rbx1 in hyperplasia/adenomas tissues with focused on few substrates, known to act as the tumor suppressors. Indeed, IHC staining showed that the levels of p21 and Foxo1, two tumor suppressors were increased substantially, whereas the levels of p27 and Nrf2 were not elevated in *Rbx1*‐null tumor tissues (Figure 1G). Thus, tumor growth inhibition by *Rbx1*‐deletion is also likely attributable to accumulation of cell cycle inhibitor p21 and apoptosis inducer Foxo1, but not p27 and Nrf2. These results also suggest that the specificity of Rbx1 substrates is in a context dependent manner.

By using the same *Kras^G12D^*‐lung in vivo tumorigenesis model, we previously reported that *Sag*/*Rbx2* deletion significantly inhibited lung tumorigenesis induced by *Kras^G12D^* via causing accumulation of p21, p27, pI*κ*b (to inactivate Nf‐*κ*b), and inactivation of mTorc1.^5^ Here we showed that *Rbx1* deletion also suppressed *Kras^G12D^*‐induced lung tumorigenesis by causing accumulation of p21 and Foxo1, but not of p27 and Nrf2, along with inactivation of both Mapk and mTorc1 pathways (Figure 1G). Thus, two RING proteins have unique as well as overlapping activities with functional nonredundant in regulation of *Kras^G12D^*‐induced lung tumorigenesis, in analogues to their role in mouse embryogenesis.^2^ Given either family member is required for Kras^G12D^‐induced lung tumorigenesis, our studies support the notion that targeting CRL E3 ligase could be an effective approach for the treatment of lung cancer associated with *Kras^G12D^* mutation.

In summary, our study fits the following working model: during lung tumorigenesis triggered by *Kras^G12D^*, Rbx1 collaborates with Kras^G12D^ by promoting the ubiquitylation and degradation of tumor suppressors (e.g., p21 and Foxo1) as well as activating Mapk and mTorc pathways, leading to enhanced tumor progression. Upon *Rbx1*‐deletion, these growth‐promoting effect was abrogated, leading to suppression of tumor progression and extension of mouse life‐span (Figure 1H). Thus, like its family member *Sag*,^5^ *Rbx1* is also a *Kras*‐cooperative gene, and CRL E3s appears to be the valid targets for anti‐lung cancer therapy.

## AUTHOR CONTRIBUTIONS

Y. L. and D. W. performed experiments and analyzed data; H. L. performed bioinformatics analysis; D. W. and Y. S wrote the manuscript; Y. S. conceived and supervised the project, and analyzed data. All authors have read and approved the final manuscript.

## CONFLICT OF INTEREST STATEMENT

The authors declare no competing interests.

## FUNDING INFORMATION

This project was supported by National Key R&D Program of China (2021YFA1101000 to Y. S.), National Natural Science Foundation of China (U22A20317, 92253203 to Y. S. and 82172699 to D. W.), and Zhejiang Provincial Natural Science Foundation of China (LD22H300003 to Y. S.).

### ETHICS STATEMENT

All animal experiments were approved by the Animal Ethics Committee of Zhejiang University, animal care was provided in accordance with the principles and procedures by the regulatory standards at Zhejiang University Laboratory Animal Center (Number: ZJU20160356; date: March 2, 2016).

## Supporting information

Supporting Information

## Data Availability

The data are available from the corresponding author upon reasonable request.
