# Supplementary material for: Inactivation of Rbx1 E3 ligase suppresses KrasG12D ‐driven lung tumorigenesis
Source: MedComm (2020). 2023 Jul 18;4(4):e332. doi: 10.1002/mco2.332 (PMC10353525; doi:10.1002/mco2.332)
Supplement: Supplementary file 1 — Supporting Information [file MCO2-4-e332-s001.pdf]

Material and methods

1. Mice

LSL-*Kras*^{G12D} (Lox-STOP-Lox-*Kras*^{G12D}) mouse model was described previously.[1] The *Rbx1*$^{fl/fl}$ mice were generated and characterized as described.[2] The LSL-*Kras*^{G12D}; *Rbx1*$^{fl/fl}$ compound experimental mice were generated by inter-crossing. All mice were maintained in SPF conditions.

2. Ad-Cre infection of mouse lung

To activate *Kras*^{G12D} (control) or to activate *Kras*^{G12D} with simultaneously inactivation of *Rbx1* (experiment) in mouse lung, the intratracheally administration of Ad-Cre was performed. Briefly, mice at age of 8 to 10-week-old were anesthetized with Pentobarbital via intraperitoneal injection. Ad-Cre adenovirus (purchased from Hanbio Co. LTD, Shanghai, China) were administered by a gel-loading tip at the dose of 3×10^7 pfu in 50μl DMEM medium.

3. H&E staining and immuno-histochemical staining

At 12 weeks post Ad-Cre administration, mice were sacrificed and the lung organs were harvested, fixed in 10% formalin, embedded in paraffin and sectioned (4 μm), followed by H&E staining and examination under a microscope.

For immunohistochemistry, the sections were deparaffinized in xylene and rehydrated through graded ethanol, antigen retrieval was performed for 20 min at 95 °C with 0.1% sodium citrate buffer (pH 6.0). Following quenching of endogenous peroxidase activity with 3% $H_2O_2 \cdot dH_2O$ and blocking of non-specific binding with 1% bovine serum albumin buffer, sections were incubated overnight at 4 °C with antibody. Following several washes, the sections were treated with HRP conjugated secondary antibody for 30 min at room temperature, and stained with 0.05% 3, 3-diaminobenzidine tetrahydrochloride (DAB). The antibodies used were purchased from Servicebio for Ki67 (GB111499), p27 (GB13154-1), and Nrf2 (GB13148); and all others were purchased from Cell Signaling Technology: p4e-bp (2855S), pErk (4376S), Active Caspase-3 (9661S), Lc3b, (2775S), p21 (2946S), and Foxo1 (2880S).

References：

1. Li H, Tan M, Jia L, et al. Inactivation of SAG/RBX2 E3 ubiquitin ligase suppresses KrasG12D-driven lung tumorigenesis. *J Clin Invest*. 2014; **124**(2): 835-846.

2. Wu D, Li H, Liu M, et al. The Ube2m-Rbx1 neddylation-Cullin-RING-Ligase proteins are essential for the maintenance of Regulatory T cell fitness. *Nat Commun*. 2022; **13**(1): 3021.
